# scMCs: a framework for single-cell multi-omics data integration and multiple clusterings

**DOI:** 10.1093/bioinformatics/btad133

**Published:** 2023-03-17

**Authors:** Liangrui Ren, Jun Wang, Zhao Li, Qingzhong Li, Guoxian Yu

**Affiliations:** School of Software, Shandong University, Jinan 250101, Shandong, China; Joint SDU-NTU Centre for Artificial Intelligence Research, Shandong University, Jinan 250101, China; Joint SDU-NTU Centre for Artificial Intelligence Research, Shandong University, Jinan 250101, China; College of Computer Science, Zhejiang University, Hangzhou 310058, China; School of Software, Shandong University, Jinan 250101, Shandong, China; Joint SDU-NTU Centre for Artificial Intelligence Research, Shandong University, Jinan 250101, China; School of Software, Shandong University, Jinan 250101, Shandong, China; Joint SDU-NTU Centre for Artificial Intelligence Research, Shandong University, Jinan 250101, China

## Abstract

**Motivation:**

The integration of single-cell multi-omics data can uncover the underlying regulatory basis of diverse cell types and states. However, contemporary methods disregard the omics individuality, and the high noise, sparsity, and heterogeneity of single-cell data also impact the fusion effect. Furthermore, available single-cell clustering methods only focus on the cell type clustering, which cannot mine the alternative clustering to comprehensively analyze cells.

**Results:**

We propose a single-cell data fusion based multiple clustering (scMCs) approach that can jointly model single-cell transcriptomics and epigenetic data, and explore multiple different clusterings. scMCs first mines the omics-specific and cross-omics consistent representations, then fuses them into a co-embedding representation, which can dissect cellular heterogeneity and impute data. To discover the potential alternative clustering embedded in multi-omics, scMCs projects the co-embedding representation into different salient subspaces. Meanwhile, it reduces the redundancy between subspaces to enhance the diversity of alternative clusterings and optimizes the cluster centers in each subspace to boost the quality of corresponding clustering. Unlike single clustering, these alternative clusterings provide additional perspectives for understanding complex genetic information, such as cell types and states. Experimental results show that scMCs can effectively identify subcellular types, impute dropout events, and uncover diverse cell characteristics by giving different but meaningful clusterings.

**Availability and implementation:**

The code is available at www.sdu-idea.cn/codes.php?name=scMCs.

## 1 Introduction

The advancement of single-cell sequencing techniques assists researchers to simultaneously obtain multiple omics data, which in return more precisely characterize the joint regulatory mechanism of multiple molecules (Luecken and Theis 2019). Specifically, single-cell RNA-sequencing (scRNA-seq) quantifies the mRNA abundance of genes in each cell, while single-cell Assay for Transposase-Accessible Chromatin using sequencing (scATAC) characterizes the openness of cis-regulatory elements in nearby genes (Zhu et al. 2020). The joint analysis of scRNA-seq and scATAC data can strength key genetic information of different omics, and decipher gene regulatory relationships related with cellular heterogeneity (Macaulay et al. 2017; Hao et al. 2021).

Although the integration of single-cell multi-omics data can facilitate the study of complex biological information, the inherent characteristics of single-cell data, such as high sparsity, noise, and dimensionality mismatch, bring great computational and analytical challenges. Researchers have been developing single-cell multi-omics integration methods by leveraging machine learning and bio-analytical techniques. A line of methods build on non-negative matrix factorization or principal component analysis to integrate single-cell multi-omics data and resolve cellular heterogeneity (Duren et al. 2018; Welch et al. 2019; Argelaguet et al. 2020; Ma et al. 2022). But these shallow methods mostly project multi-omics data into a shared latent space and ignore omics-specific information. Furthermore, linear models disregard non-linear geometries of multi-omics data. Manifold alignment methods aim to align embedded low-dimensional manifolds of different omics data and characterize intrinsic cellular structures (Liu et al. 2019; Cao et al. 2021). Although these alignment-based methods can capture non-linear geometries across multi-omics data, they suffer a high time complexity $O(N^{3})$ (*N* is the number of samples), which limits their applications.

By the virtue of expressive feature extraction capability, deep-learning methods have emerged as the mainstream technique for single-cell data analysis (Tian et al. 2019; Xiong et al. 2019; Liu et al. 2021a). Recently, Zuo and Chen (2021) proposed single-cell Multimodal Variational AutoEncoder (scMVAE) to integrate scRNA-seq and scATAC data. Specifically, scMVAE combines probabilistic Gaussian mixture models with three different joint learning strategies to explore latent features that can characterize multi-omics data. But merely embedding different omics data into the same latent space may lose the specificity of individual omics. Unlike scMVAE, Deep Cross-omics Cycle Attention (DCCA) (Zuo et al. 2021) uses different deep generative networks to model the scRNA-seq and scATAC data, then applies attention-transfer to explore the regulations between different omics and cell heterogeneity.

The aforementioned deep methods still have some issues. First, most of them focus on a shared representation, but disregard the omics individuality, and cannot integrate different levels of biological features to learn a more discriminative representation for data imputation and cell clustering. Furthermore, contemporary single-cell clustering methods only aim at one clustering of cell types. In practice, cells can also be clustered by other biological characteristics, such as cell functions or states, and these biological characteristics can be regulated by gene expression. Existing methods cannot sufficiently integrate and merge the genetic information from different omics to reveal potential alternative clusterings with diversity and high quality, while these multiple clusterings can reveal the different roles and characteristics of cells from different perspectives.

**Figure 1:**
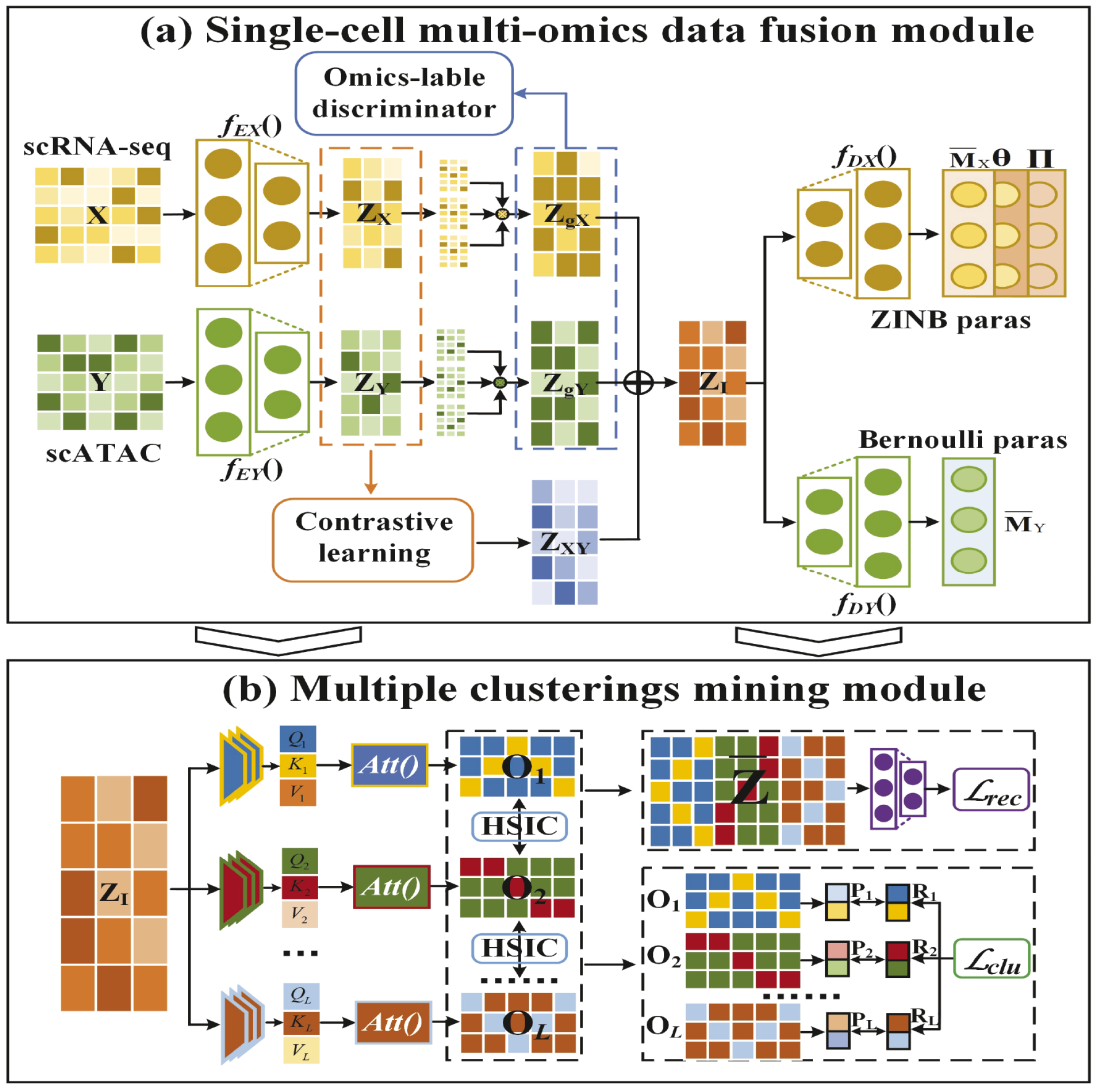
Framework overview of scMCs for single-cell multi-omics data fusion and multiple clusterings. (a) scMCs firstly projects scRNA-seq data X and scATAC data Y into different low-dimensional spaces $Z_{X}$ and $Z_{Y}$. Next, it utilizes the attention mechanism and omics-label discriminator to extract the omics individuality $Z_{gX}$ and $Z_{gY}$, and uses contrastive learning mechanism to capture omics commonality $Z_{XY}$. After that, scMCs fuses the omics individuality and commonality to obtain an informative co-embedding matrix $Z_{I}$ for clustering, and learns the parameter representations $\left\{{\bar{M}}_{X},\Theta ,\Pi \right\}$ of ZINB distribution and $\left\{{\bar{M}}_{Y}\right\}$ of Ber. (b) scMCs projects $Z_{I}$ into different subspaces ${\left\{O_{l}\right\}}_{l=1}^{L}$, leverages the redundancy control constraint and clustering loss $L_{\mathrm{clu}}$ to enhance the diversity and quality among them for generating multiple clusterings ${\left\{R_{l}\right\}}_{l=1}^{L}$ in an end-to-end manner. Besides, scMCs minimizes the reconstruction loss $L_{\mathrm{rec}}$ to ensure the consistency of feature information

To address these challenges, we propose a method called scMCs and present the conceptual framework in Fig. 1. The main idea of our solution is to design an information extraction and fusion module to finely process the individuality and commonality learned from heterogeneous omics, and construct a more comprehensive and informative representation for single-cell multi-omics data fusion, clustering, and multiple clustering. Specifically, scMCs uses the omics-independent deep autoencoders to learn the low-dimensional representation of each omics, and utilizes the attention mechanism and omics-label discriminator to capture the omics individuality. Meanwhile, scMCs utilizes the contrastive learning strategy to capture the commonality, and fuses the individuality and commonality features into a compact co-embedding representation for cell clustering and data imputation. To uncover the potential alternative clusterings in multi-omics data, scMCs applies multi-head attention mechanism (Vaswani et al. 2017) on the co-embedding representation to generate multiple salient subspaces, and reduce the redundancy between subspaces. Meanwhile, scMCs optimizes a Kullback–Leibler (KL) divergence-based clustering loss in each salient subspace and generates different high quality clusterings in an end-to-end framework.

## 2 Materials and methods

The framework overview of scMCs is shown in Fig. 1, where Fig. 1a aims at multi-omics data fusion and cell clustering; and Fig. 1b targets to explore multiple clusterings with quality and diversity embedded in multi-omics data. The technical details of scMCs are presented below.

### 2.1 Multi-omics data encoder for individuality

With the increasing complexity of single-cell data, researchers have merged deep learning with single-cell data clustering (Liu et al. 2021a). As a classical neural network, autoencoder can map high-dimensional data into a low-dimensional representation space while ignoring noise and outliers. Given that, we separately use autoencoders to map single-cell multi-omics data into their respective non-linear embedding spaces, thereby preserving the individuality, resisting noises and outliers.

Let $X\in R^{N\times D_{X}}$ and $Y\in R^{N\times D_{Y}}$ be the normalized scRNA-seq data and scATAC data, where *N* is the number of samples, $D_{X}$ and $D_{Y}$ are the number of features. scMCs firstly employs two independent encoders $f_{EX}()$ and $f_{EY}()$ to learn respective *d*-dimensional feature representations $\{Z_{X},Z_{Y}\}\in R^{N\times d}$: where *d* is the dimension of embedding space; $Z_{X}$ is the latent low-dimensional representation of cells and genes in scRNA-seq data, while $Z_{Y}$ encodes the latent patterns between cells and peaks in scATAC data.


(1) $$Z_{X}=f_{EX}(X),Z_{Y}=f_{EY}(Y),$$

To extract the individuality and explore the complementary information among different omics, we incorporate the attention mechanism and omics-label discriminator into the encoder module. Concretely, scMCs defines two normalized attention score matrices as: where the elements in $A_{X}$ and $A_{Y}$ quantify the similarity of a pair of cells for different omics. Softmax(⋅) normalizes the weight to [0, 1] to avoid modeling negative correlations, it also helps to prevent the local optimal problem caused by too large weights of some cells. With the normalized attention scores, we reorganize the low-dimensional representations by considering the similarity among cells:


(2) $$A_{X}=\mathrm{softmax}(\frac{Z_{X}{\left(Z_{X}\right)}^{T}}{\sqrt{d}}),\;A_{Y}=\mathrm{softmax}(\frac{Z_{Y}{\left(Z_{Y}\right)}^{T}}{\sqrt{d}}),$$

(3) $$Z_{gX}=A_{X}Z_{X},\;Z_{gY}=A_{Y}Z_{Y}.$$

The attention mechanism plays important roles in the encoding module. On the one hand, it measures the importance of biological signals in the intrinsic feature spaces of different omics, and extracts omics individuality; on the other hand, it explores the similarity between cells and enables to explore the representation relationship between cells and features from a global perspective.

In supervised learning tasks, labels can indicate the class or identity of the samples. Given that, omics labels can be used as the supervised signals to extract individual features of each omics. Here, we explicitly define the omics labels, i.e. cells from the same omics are labeled as one type. Next, we design an omics-label discriminator to further enhance the quality of individuality in $Z_{gX}$ and $Z_{gY}$. The discriminator loss is defined as: where CE is the cross-entropy loss, $P\in {\left\{0,1\right\}}^{2N\times K}$ is the true omics-label matrix, where *K* is the number of omics; $f_{\mathrm{dis}}()$ is the omics-label predictor, which is a fully connected neural network with two layers.


(4) $$L_{\mathrm{dis}}=CE(P,f_{\mathrm{dis}}(Z_{gX},Z_{gY})),$$

### 2.2 Cross-omics contrastive learning for commonality

The attention layers and omics-label discriminator may induce the model to pay more attention to individual features or noises of each omics, which is not conducive to data fusion and cell clustering. Furthermore, individual features only unilaterally characterize the complementarity between omics, while the cross-omics consistent (shared) information can reflect the commonality between omics, which is important for a consistent clustering with high quality. Existing methods (i.e. MOFA+, CoNMF, and scMVAE) mainly concatenate the multi-omics data and project them into a common low-dimensional representation to explore the shared information. However, due to the sparsity and high dimensionality of different omics, the resulting representation may be of low quality. Although DCCA (Zuo et al. 2021) uses different deep generative autoencoders and the attention-transfer to link multi-omics, it pays more attention to the knowledge learned from scRNA-seq but lacks attention to scATAC. To extract the compact commonality features between different omics, we introduce the cross-omics contrastive learning strategy (Liu et al. 2021b) to extract shared knowledge from scRNA-seq and scATAC data for fusion.

As a novel self-supervised learning paradigm, the core theory of contrastive learning is to maximize the consistency by maximizing the mutual information between different views (Chen and Geng 2021). In this way, we can obtain more informative embedded features by maximizing the information entropy, and avoid the simple solution of assigning all samples to the same cluster. The details of learning commonality are as follows:

- *Feature multilayer perceptron (MLP)*: To eliminate the influence of heterogeneity and ensure the semantic consistency of $Z_{X}$ and $Z_{Y}$, scMCs maps $Z_{X}$ and $Z_{Y}$ into one latent semantic space via a shared feature MLP:
  (5) $$Q_{X}=f_{\mathrm{MLP}}(Z_{X}),\;Q_{Y}=f_{\mathrm{MLP}}(Z_{Y}),$$
  where $\{Q_{X}$, $Q_{Y}\}\in R^{N\times d}$ are low-dimensional embedding representations of X and Y with similar semantics.
- *Cross-omics contrastive learning*: In the latent space parameterized by $f_{\mathrm{MLP}}$, we optimize the contrastive loss between $Q_{X}$ and $Q_{Y}$ to learn the commonality representation as:
  (6) $$L_{cl}=-(I(Q_{X},Q_{Y})+\epsilon (H(Q_{X})+H(Q_{Y}))),$$
  where I(⋅) denotes the mutual information, H(⋅) is the information entropy, and ϵ is a weight parameter. Finally, scMCs integrates the consistent representations as follows:
  (7) $$Z_{XY}=f_{XY}(Q_{X},Q_{Y}),$$
  where $Z_{XY}$ encodes the commonality of different omics, $f_{XY}$ is a fully connected neural network with two layers.

### 2.3 Multi-omics data fusion and imputation for clustering

As discussed, scMCs can learn two latent representations $Z_{gX}$ and $Z_{gY}$ to encode omics individuality, and a latent representation $Z_{XY}$ to encode commonality, which are key factors for clustering and imputing single-cell multi-omics data. Here, we perform an element-wise sum operation with scale parameters $\lambda_{x}$ and $\lambda_{y}$ to aggregate them, and generate a more discriminative co-embedding representation $Z_{I}$:


(8) $$Z_{I}=Z_{XY}+\lambda_{x}Z_{gX}+\lambda_{y}Z_{gY}.$$

A simple solution to optimize the co-embedding representation $Z_{I}$ is to use different MLP as decoders to reconstruct each omics. However, frequent dropout events may seriously affect the quality of $Z_{I}$ and lead to inaccurate clustering results. In practice, we can impute the dropout events and utilize the imputed data feedback to optimize $Z_{I}$, further enhancing the accuracy of key genetic features. Previous studies show that scRNA-seq data often have the characteristics of discreteness, variance greater than the mean and high sparsity (Risso et al. 2018). Nonetheless, some studies report the zero-inflated negative binomial (ZINB) probability distribution can account for these characteristics (Eraslan et al. 2019). Therefore, we propose a ZINB model based decoder network to explore the global probabilistic structure of scRNA-seq data. Mathematically, ZINB is defined with the mean ($\mu_{x}$) and dispersion (θ) parameters of the negative binomial distribution and a coefficient (π) that describes the probability of dropout events: where x is a vector from the original scRNA-seq data.


(9) $$NB(x;\mu_{x},\theta )=\frac{\Gamma (x+\theta )}{\Gamma (\theta )}{\left(\frac{\theta }{\theta +\mu_{x}}\right)}^{\theta }{\left(\frac{\mu_{x}}{\theta +\mu_{x}}\right)}^{x},$$

(10) $$\mathrm{ZINB}(x;\pi ,\mu_{x},\theta )=\pi \zeta_{0}(x)+(1-\pi )NB(x;\mu_{x},\theta ),$$

In details, the ZINB-based decoder estimates the parameters $\left\{\pi ,\mu_{x},\theta \right\}$ based on $Z_{I}$ through three different fully connected layers as follows: where $\left\{\Pi ,{\bar{M}}_{X},\Theta \right\}$ is the matrix form of $\left\{\pi ,\mu_{x},\theta \right\}$; $f_{DX}$ is a decoder with fully connected layer; $W_{\pi }$, $W_{\mu_{x}}$, and $W_{\theta }$ are three learnable parameter matrices. The activation function of Π is sigmoid() because the dropout probability is between 0 and 1. In addition, since the mean and dispersion parameters are non-negative, the exponential function exp() is selected as the activation function for ${\bar{M}}_{X}$ and Θ.


(11) $$\Pi =\mathrm{sigmoid}(f_{DX}(Z_{I},W_{\pi })),$$

(12) $${\bar{M}}_{X}=\text{exp}(f_{DX}(Z_{I},W_{\mu_{x}})),\;\Theta =\text{exp}(f_{DX}(Z_{I},W_{\theta })),$$

Different from the traditional mean squared error loss-based autoencoder, the loss function of ZINB-based decoder network is the negative log of the ZINB likelihood:


(13) $$L_{\mathrm{ZINB}}=-\log(\mathrm{ZINB}(X|\Pi ,{\bar{M}}_{X},\Theta )).$$

Considering the extremely sparse and nearly binary nature of scATAC data, we use a Bernoulli distribution (Ber)-based decoder network to model scATAC data: where y is a vector from the original scATAC data; $\mu_{y}$ is the mean parameters of Ber. The Bernoulli-based decoder estimates $\mu_{y}$ based on $Z_{I}$ through a fully connected layer with sigmoid() as activation function: where ${\bar{M}}_{Y}$ is the matrix form of $\mu_{y}$ and $W_{\mu_{y}}$ is the weight parameter matrix. Finally, the Bernoulli-based autodecoder can be optimized by the cross-entropy loss:


(14) $$\mathrm{Ber}(y;\mu_{y})=y\;\text{log}(\mu_{y})+(1-y)\log(1-\mu_{y}),$$

(15) $${\bar{M}}_{Y}=\mathrm{sigmoid}(f_{DY}(Z_{I},W_{\mu_{y}})),$$

(16) $$L_{\mathrm{Ber}}=CE(Y,{\bar{M}}_{Y}).$$

To pursue a more discriminative and informative co-embedding representation that incorporates individuality and commonality of multi-omics data, we unify the objective of imputing the scRNA-seq data and scATAC data, predicting the omics labels, and cross-omics contrastive learning loss as follows: where $\Phi_{1}$ denotes the network parameters, $\alpha_{1}$, $\alpha_{2}$, and $\alpha_{3}$ are three scalar parameters to constrain $L_{\mathrm{Ber}}$, $L_{\mathrm{dis}}$, and $L_{cl}$. By optimizing Equation (17), the individual and shared feature representations can be learned from multi-omics data, and they can be merged into an informative co-embedded representation for clustering and multiple clustering.


(17) $$\begin{array}{c} L_{1}=\underset{\Phi_{1}}{\mathrm{argmin}}((-\log(\mathrm{ZINB}(X|\Pi ,{\bar{M}}_{X},\Theta )))+\alpha_{1}CE(Y,{\bar{M}}_{Y})\\ +\alpha_{2}CE(P,f_{\mathrm{dis}}(Z_{gX},Z_{gY}))\\ +\alpha_{3}(-(I(Q_{X},Q_{Y})+\epsilon (H(Q_{X})+H(Q_{Y}))))), \end{array}$$

### 2.4 Multiple clusterings mining module

Contemporary single-cell multi-omics analysis methods mainly aim to integrate cross-omics shared features to find an optimal cell division pattern, which ignores other potential important patterns. Due to the multiplicity of multi-omics data, different cell clustering patterns, such as cell type clustering or cell state clustering, can co-exist. Unlike traditional multi-view clustering methods that can only discover a single clustering, multi-view multiple clustering can incorporate the omics consistent and specific features and simultaneously generate multiple meaningful and non-redundant clusterings, which help us to divide cells from different perspectives and explain the cell heterogeneity. Different from subspace clustering that finds one clustering with clusters spanned in different subspaces, multiple clustering explores alternative clusterings in different subspaces. To more comprehensively mine single-cell multi-omics data, scMCs introduces another module (as illustrated in Fig. 1b), and proposes to sufficiently utilize the omics individuality and commonality to explore alternative clusterings embedded in the multi-omics data.

A naive idea to generate multiple clusterings is to define multiple embedding subspaces based on the original or imputed data. However, the resulting embeddings/clusterings may largely overlap, due to the characteristics of high noise and sparsity of single-cell data. Here, scMCs uses $Z_{I}$ to generate different salient subspaces for its compactness with informative features. Specifically, it applies multi-head attention on $Z_{I}$ to generate *L* salient heads ${\left\{O_{l}\right\}}_{l=1}^{L}$, which capture different perspectives of $Z_{I}$, and thus generate *L* salient subspaces. The *l*-th head $O_{l}\in R^{N\times m}$ is calculated as: where $\left\{Q_{l},K_{l},V_{l}\right\}$ are the linear transformations of $Z_{I}$ with respect to different parameters $\left\{W_{l}^{Q},W_{l}^{K},W_{l}^{V}\right\}$, *m* is the dimension of each head. It is worth noting that projecting $Z_{I}$ with different parameters can theoretically control the difference between heads, and thus help to generate diverse subspaces and clusterings.


(18) $$O_{l}=\mathrm{softmax}(\frac{Q_{l}K_{l}^{T}}{\sqrt{m}})V_{l},$$

To ensure the consistency between subspace features and $Z_{I}$, we concatenate all the heads as $\bar{Z}=\text{concat}(O_{1},\ldots ,O_{L})$ and decode $\bar{Z}$ toward $Z_{I}$ with the following reconstruction loss:


(19) $$L_{\mathrm{rec}}=||Z_{I}-{\bar{Z}}_{I}|{|}_{2}^{2},\;{\bar{Z}}_{I}=f_{\bar{\Theta }}(\bar{Z}).$$

One key concern of multiple clusterings is how to reduce the redundancy between clusterings. Although with different linear transformation parameters, the multi-head attention may still produce redundant subspaces. Here, we leverage the Hilbert Schmidt Independence Criterion (HSIC) (Gretton et al. 2005) to quantify the dependency between heads, which also approximately measures the redundancy between subspaces and clusterings. Theoretically, HSIC quantifies the dependency between two head $O_{l}$ and $O_{l^{\prime }}$ based on the norm of the cross-covariance operator. It can simultaneously measure the linear and non-linear dependency between representations. The larger the HSIC value, the larger the dependency between them is. The empirical HSIC is computed as: where Tr(⋅) is the trace norm, $U_{l}=O_{l}^{T}O_{l}$ is the Gram matrix, $H=I_{m}-\frac{1}{m}11^{T}$ centers the Gram matrices to have zero mean. Mathematically, the dependency among *L* heads is computed as: where ${\tilde{U}}_{l}={\left(m-1\right)}^{-2}\sum_{l=1,l\neq l^{\prime }}^{L}HU_{l^{\prime }}H$. Minimizing Equation (21) penalizes the dependency among *L* heads, and reduces the redundancy between different subspaces and clusterings therein.


(20) $$\mathrm{HSIC}(O_{l},O_{l^{\prime }})=\frac{1}{{\left(m-1\right)}^{2}}Tr(U_{l}HU_{l^{\prime }}H),$$

(21) $$\begin{array}{cc} L_{\mathrm{red}} & =\sum_{l=1,l\neq l^{\prime }}^{L}HSIC(O_{l},O_{l^{\prime }})=\sum_{l=1}^{L}Tr(O_{l}{\tilde{U}}_{l}O_{l}^{T}) \end{array},$$

Another concern of multiple clusterings is how to maintain the quality of each clustering, which describes the compactness within clusters and the separation between clusters. Here, we propose to learn *L* sets of cluster centers ${\left\{\Omega_{l}\right\}}_{l=1}^{L}$ in *L* subspaces ${\left\{O_{l}\right\}}_{l=1}^{L}$, where $\Omega_{l}=\{\omega_{l}^{1},\omega_{l}^{2},\ldots ,\omega_{l}^{J_{l}}\}$ indicates that $O_{l}$ has $J_{l}$ cluster centers.

To optimize the cluster centers in each subspace, we utilize a KL divergence loss to enhance the association between similar cells. Specifically, we measure the pairwise similarity between the sample point $o_{l}^{i}$ and centroid $\omega_{l}^{j}$ in $O_{l}$ as follows: where $p_{ij}\in P_{l}$ is the probability of assigning sample i (1≤i≤N) to cluster $j\;(1\leq j\leq J_{l})$. Equation (22) uses a *t*-distribution constraint to optimize the distance between samples and cluster centers, which can generate larger gradients for dissimilar samples to prevent clustering them together.


(22) $$p_{ij}=\frac{{\left(1+||o_{l}^{i}-\omega_{l}^{j}|{|}^{2}\right)}^{-1}}{\sum_{j}{\left(1+||o_{l}^{i}-\omega_{l}^{j}|{|}^{2}\right)}^{-1}},$$

To further optimize the cluster centers and strengthen the affinity between similar samples, we introduce an auxiliary target distribution $R_{l}$ to refine the clusters in each clustering by learning their high-confidence assignments (Xie et al. 2016), and its elements can be computed as:


(23) $$r_{ij}=\frac{p_{ij}^{2}/\sum_{i}p_{ij}}{\sum_{j}\left(p_{ij}^{2}/\sum_{i}p_{ij}\right)}.$$

Theoretically, $R_{l}$ can improve the compactness between similar samples, while paying less attention to dissimilar ones. In addition, it balances the contribution of each cluster center through normalization, and avoids the clustering distortion caused by a larger cluster.

Based on these two similarity distribution functions, we can define the clustering loss among *L* heads as:


(24) $$L_{\mathrm{clu}}=\sum_{l=1}^{L}KL(P_{l}||R_{l})=\sum_{l=1}^{L}\sum_{i=1}^{N}\sum_{j=1}^{J_{l}}p_{ij}\;\text{log}\;\frac{p_{ij}}{r_{ij}}.$$

To generate multiple diverse subspaces from $Z_{I}$ and explore high quality clusterings therein, we unify the objective of reconstruction loss, redundancy between subspaces, and clustering loss as follows: where $\Phi_{2}$ is the network parameters, $\beta_{1}$ and $\beta_{2}$ are two scalar parameters to balance the diversity and quality. By optimizing Equation (25), we can find multiple salient subspaces from the co-embedding representation $Z_{I}$, and also generate multiple clusterings with high quality therein in an end-to-end manner. When updating *l*-th clustering $C_{l}$, the label assigned to *i*-th sample can be made as $c_{i}=\text{argmax}r_{ij},C_{l}={\left\{c_{i}\right\}}_{i=1}^{N}$. If we fix L=1, the redundance control term in Equation (25) is disregarded, then, we can learn an embedded representation $O_{1}$ of multiple omics and discover the single clustering therein.


(25) $$\begin{array}{c} L_{2}=\underset{\Phi_{2}}{\text{argmin}}(||Z_{I}-{\bar{Z}}_{I}|{|}_{2}^{2}+\beta_{1}\sum_{l=1}^{L}Tr(O_{l}{\tilde{U}}_{l}O_{l}^{T})\\ +\beta_{2}\sum_{l=1}^{L}KL(P_{l}||R_{l})), \end{array}$$

## 3 Results

### 3.1 Experiment setup

**Datasets:** scMCs is a flexible framework that can integrate different single-cell omics data. In the experiments, we mainly evaluate the performance of scMCs by jointly modeling the scRNA-seq data and scATAC data. We collect four preprocessed single-cell multi-omics data with paired profiles from a previous study (Zuo et al. 2021): (i) CellMix with 1047 cells is downloaded from GEO (D1, GSE126074), in which the chromatin accessibility and gene expression in each single-cell are simultaneously co-assayed using the SNARE-seq; (ii) PBMC_3K (D2) with 3012 cells is downloaded from 10X Genomics; (iii) Mouse_skin downloaded from GEO (D3, GSE140203) contains 34 774 cells, and it is derived from adult mouse skin by SHARE-seq. (iv) AdBrain with 10 309 cells is downloaded from GEO (D4, GSE126074), in which the chromatin accessibility and gene expression in each single-cell are derived from the adult mouse cerebral cortex. We use the Signac package (Stuart et al. 2021) to preprocess AdBrain dataset, and retain the top 5000 highly variable-genes of scRNA-seq data and 52 818 peaks of the scATAC data.

**Evaluation protocols:** For ‘single clustering’, *k*-means is applied to cluster the cells based on the learned low-dimensional co-embedding representation $Z_{I}$. Then, we use Normalized Mutual Information (NMI) and Adjusted Rand Index (ARI) to evaluate the clustering performance. The range of NMI and ARI are both [0,1], and a higher value indicates a better clustering performance. For ‘multiple clusterings’, we use the NMI and Jaccard Index (JI) to measure the overlap between different clusterings, and Silhouette Coefficient (SC) and Dunn Index (DI) to evaluate the quality of each clustering.

**Comparing baselines:** We implement scMCs with the MindSpore deep learning framework and compare it against with Iv competitive single-cell multi-omics data fusion methods. (i) **JSNMF** (Ma et al. 2022) decomposes different omics data into different latent spaces, and learns the consistent information of multi-omics data through a consensus graph; (ii) **UnionCom** (Cao et al. 2020) projects multi-omics data into a common embedding space, and matches the complex non-linear features by a global scaling parameter to cluster the cells; (iii) **scMVAE** (Zuo and Chen 2021) proposes three strategies, scMVAE-PoE, scMVAE-NN, and scMVAE-Direct, to learn the joint latent features for data fusion and clustering. scMVAE-Direct concatenates raw features of each omics, scMVAE-NN combines the low-dimensional features extracted from different omics, while scMVAE-PoE uses the product of experts framework to estimate a joint posterior distribution; and (iv) **DCCA** (Zuo et al. 2021) projects different omics into their corresponding low-dimensional spaces, and uses the ‘Teacher-student’ mechanism to fuse multi-omics data. The experimental configurations of these compared methods are given in Supplementary Table S1.

### 3.2 Cell clustering and visualization

Table 1 summarizes the clustering performance of scMCs and other baselines on four datasets. Each method repeats five times to take the average and variance, and the bold fonts indicate the best result. UnionCom is too time-consuming on large datasets, so its results on Mouse_skin are not reported. scMCs performs well on the four datasets in terms of NMI and ARI, and the clustering results are statistically better than other methods in most cases. Other important observations are as follows:

**Table 1.** Performance of single clustering of compared methods on different datasets.^a^

		JSNMF	UnionCom	scMVAE-PoE	scMVAE-NN	scMVAE-Direct	DCCA	scMCs
D1	NMI	0.262 ± 0.003•	0.704 ± 0.004•	0.852 ± 0.002•	0.817 ± 0.001•	0.811 ± 0.000•	0.619 ± 0.000•	**0.907 ** ± **0.000**
	ARI	0.196 ± 0.003•	0.670 ± 0.005•	0.839 ± 0.001•	0.819 ± 0.000•	0.811 ± 0.001•	0.513 ± 0.001•	**0.939 ** ± **0.000**
D2	NMI	0.416 ± 0.000•	0.606 ± 0.000°	0.603 ± 0.002°	**0.611 ** ± **0.001**°	0.505 ± 0.002•	0.414 ± 0.000•	0.534 ± 0.001
	ARI	0.284 ±0.004•	0.400 ± 0.001•	0.452 ± 0.007•	0.447 ± 0.003•	0.441 ± 0.004•	0.404 ± 0.000•	**0.596 ** ± **0.000**
D3	NMI	0.140 ± 0.000•		0.334 ± 0.000•	0.331 ± 0.000•	0.294±0.001•	0.265 ± 0.000•	**0.433 ** ± **0.000**
	ARI	0.087 ± 0.000•		0.250 ± 0.000•	0.260 ± 0.000°	0.232 ± 0.002•	0.250 ± 0.001•	**0.260 ** ± **0.000**
D4	NMI	0.269 ± 0.000•	0.305 ± 0.001•	0.325 ± 0.001•	0.287 ± 0.005•	0.273±0.003•	0.296 ± 0.003•	**0.510 ** ± **0.000**
	ARI	0.194 ± 0.001•	0.248 ± 0.005•	0.268 ± 0.001•	0.164 ± 0.002•	0.125 ± 0.002•	0.197 ± 0.001•	**0.554 ** ± **0.001**

a • /° indicates whether scMCs is superior/inferior to the other method, with statistical significance checked by pairwise *t*-test at 95% level. The best results are highlighted in bold font.

- **scMCs versus JSNMF:** JSNMF more focuses on the linear and shared features, but overlooks the individual features of each omics. In addition, it neglects the impact of dropout events. Thus, it has a poor clustering performance in most cases. In contrast, scMCs can learn the omics individuality and commonality to joint optimize the co-embedding and data imputation for a better cell clustering.
- **scMCs versus UnionCom:** UnionCom not only fails to consider the influence of individual manifold features on clustering, but also cannot effectively handle the dropout events. So it loses to scMCs in most cases. Furthermore, the huge time overhead of learning the manifold topology structure also limits its application to high-dimensional data.
- **scMCs versus scMVAE:** There is a clear margin between scMVAE-PoE, scMVAE-Direct, scMVAE-NN, and scMCs, which proves the advanatages of scMCs. scMVAE-Direct has the worst performance, because concatenating the high-dimensional features can significantly increase the sparsity and complexity of data representation. scMVAE-NN performs better than scMVAE-Direct, because it explores a common representation in a more compact feature space. scMVAE-PoE learns a consistent probability distribution of multi-omics data with fewer model parameters from a global perspective, and it gives better results than scMVAE-Direct and scMVAE-NN. However, scMVAE disregards the individuality of multi-omics data for data fusion cell clustering. In constrast, scMCs not only considers shared features as key factors for a consensus cell clustering, but also the individual features.
- **scMCs versus DCCA:** Although DCCA utilizes different neural networks to project multi-omics data into different representation spaces, it loses to scMCs by a clear margin. This is because DCCA mainly focuses on the individual features of different omics data, and neglects the shared features of these omics for the consistent clustering. In contrast, scMCs simultaneously extract the shared and individual features from different omics, and fuses them into a co-embedding space, which can encode the cellular heterogeneity and find a more accurate clustering.

In addition, to illustrate the quality of $Z_{I}$, we apply uniform manifold approximation and projection (UMAP) (Becht et al. 2019) to visualize cell clustering points of scMCs and other baselines on each benchmark dataset. As shown in Supplementary Figs S1–S4, we can clearly see that scMCs has the clearest division boundaries and the lowest misclassification rate. These results also explain why scMCs achieves a better clustering performance.

### 3.3 Evaluation of data imputation

Besides accurate cell clustering, scMCs also realizes data imputation based on $Z_{I}$ using two independent deep generative decoder networks. To evaluate the quality of imputed scRNA-seq data and scATAC data, we visualize the raw data and the imputed data generated by scMCs, scMVAE-PoE, scMVAE-Direct, scMVAE-NN, and DCCA. Specifically, we project the raw data and imputed data into different 2D spaces via UMAP, and explore cell clusterings therein. Meanwhile, we also leverage NMI and ARI to evaluate the clustering given by each method.

Supplementary Figs S5–S12 report the visualization and clustering performance of each method on raw and imputed CellMix, PBMC_3K, Mouse_skin, and AdBrain, respectively. We see the NMI and ARI scores of scMCs are significantly higher than those of other baselines. The visualization results also confirm the cell clustering found by scMCs is more separated between different clusters and more compact within clusters. All these confirm that scMCs can generate an informative embedding representation $Z_{I}$, which can be used for data imputation.

In addition, to assess whether scMCs contributes to discover important biological signals, we utilize Signac to process the raw multi-omics data as well as the imputed data. Taking AdBrain as example, we report the results in Supplementary Fig. S13. Concretely, we normalize the raw scRNA-seq data and scATAC data and visualize the normalized data into a 2D space via UMAP. Then, we annotate cell types and provide the results in Supplementary Fig. S13a, where the top shows the clustering results on raw AdBrain, the bottom shows the results on imputed data. We can observe that the clusters obtained using the imputed data are more compact, and the boundaries between clusters are clearer. To study differences in gene activity across clusters, we create a gene activity matrix based on imputed scATAC data. Taking ‘L2/3 IT’, ‘L6 IT’, ‘L5 CT’, and ‘L4’ as examples, we use FindAllMarkers() function to determine the differentially expressed genes of each cell cluster, and report the results in Supplementary Fig. S13b. We can accurately identify the marker genes of different cell types using the imputed scATAC data, which prove scMCs can find out associations between genes and peaks by imputing the missing values in scATAC data. Moreover, we uncover the differentially accessible peaks between clusters using the imputed scATAC data, and report the results on four clusters in Supplementary Fig. S13c. We can observe that the peaks are significantly different among clusters, which indicates the specific accessibility in heterogeneous cell types. Overall, these results show that scMCs can achieve effective imputation of single-cell multi-omics data, reveal significant relationships between cells and genes, as well as the biological correlation between cell types and peak accessibility.

### 3.4 Evaluation of multiple clusterings

Existing single-cell data clustering methods can ‘only find one clustering pattern’ of cell types. However, with the increased multiplicity of single-cell data, there exist alternative and meaningful clusterings, which can uncover new patterns of cells at a more comprehensive way.

As shown in Fig. 1b, scMCs can project the co-embedding representation $Z_{I}$ into different salient subspaces, and find out different clusterings therein. The number of clusterings and clusters in each clustering can be specified based on the datasets or user’s expectation. If the dataset has reference label, users can refer to these labels to specify the number of clusterings and clusters. Otherwise, users can specify the expected number of alternative clusterings, next adopts widely used stable clustering techniques (Wang et al. 2021) to determine the number of clusters in each clustering, and then visualizes these clusterings or use internal evaluation metrics (i.e. SC) to determine the number of alternative clusterings and clusters therein in an explorative data mining way. In the experiments, we project $Z_{I}$ into two subspaces $\left\{O_{1},O_{2}\right\}$, and generate two clusterings $\left\{C_{1},C_{2}\right\}$. Then, we use the SC and DI to measure the overall quality of $\left\{C_{1},C_{2}\right\}$, and further compare $\left\{C_{1},C_{2}\right\}$ against the distinct ground truth $C_{t}$ of CellMix, PBMC_3K, and AdBrain. Table 2 lists the average clustering results of five independent runs of scMCs. In addition, we further evaluate the diversity between $C_{1}$ and $C_{2}$ using NMI and JI. Supplementary Fig. S14 reports the diversity (1-NMI, 1-JI) of scMCs on CellMix, PBMC_3K, and AdBrain. Concretely, NMI and JI measure the similarity between the two generated different clusterings. Hence, a larger (1-NMI or 1-JI) means these clusterings are less overlapped. Several observations can be made from these results:

**Table 2.** Diversity and quality of multiple clusterings generated by scMCs on benchmark datasets.^a^

		CellMix	PBMC_3K	AdBrain
		$C_{t}$	$C_{t}$	$C_{t}$
NMI↑	$C_{1}$	0.845	0.695	0.513
	$C_{2}$	0.365	0.204	0.289
JI↑	$C_{1}$	0.860	0.378	0.364
	$C_{2}$	0.355	0.197	0.291
SC↑	$C_{1}$	0.666	0.644	0.268
	$C_{2}$	0.599	0.826	0.579
DI↑	$C_{1}$	0.076	0.071	0.048
	$C_{2}$	0.054	0.040	0.053

a $C_{t}$ is the ground truth, while $C_{1}$ is the clustering similar to ground truth, $C_{2}$ is the other alternative clustering.

- From Table 2, we can observe that $C_{1}$ has a high similarity with the ground truth $C_{t}$, while the smaller NMI and JI values indicate that $C_{2}$ is not similar to $C_{t}$. In addition, the high SC and DI values suggest that $C_{2}$ is a potential alternative clustering with high quality.
- The results in Supplementary Fig. S14 show that there is a rather low redundancy between $C_{1}$ and $C_{2}$, this fact proves that scMCs can not only find the significant cell type clustering from the co-embedding representation $Z_{I}$, but also the other potential alternative clustering.

To verify the biological significance of $C_{1}$ and $C_{2}$, we conduct a series of downstream analyses. Taking CellMix as an example, the relevant results are shown in Supplementary Figs S15–S17. Firstly, we perform cell clustering and annotation on CellMix based on the ground truth $C_{t}$. As shown in Supplementary Fig. S15a, CellMix is divided into four cell clusters. To determine the identity of each cell cluster, we identify the marker genes in each cluster using the FindAllMarkers() function and report four differentially expressed genes in Supplementary Fig. S15b and c. According to the Cell Taxonomy database (Jiang et al. 2023), we confirm that these four genes mark four different cell lines, including H1, BJ, K562, and GM12878. In addition, Supplementary Fig. S16 provides the results of $O_{1}$ based on $C_{1}$. We find that cells in $O_{1}$ can also be clustered into four clusters. By identifying the marker genes, we identify these four cell clusters as H1, BJ, K562, and GM12878, respectively. These results can also prove that there is a cell type clustering embedded in $O_{1}$, and this is consistent with the results in Table 2.

scMCs not only can find out a clustering in accordance with the known $C_{t}$, but also other alternative ones $C_{2}$ embedded in $O_{2}$, which reveals the tissue specificity of the cells from a new perspective. Concretely, Supplementary Fig. S17a and b shows that cells in $O_{2}$ can be divided into two clusters, where the marker genes of cluster 0 are UCHL1 and CALD1, and the markers of cluster 1 are TXNIP and DDIT3. Moreover, Supplementary Fig. S17c also shows that different genes are differentially expressed in each cluster. Based on the conclusions in Cell Taxonomy database (Jiang et al. 2023) and Human Protein Atlas (Uhlen et al. 2010), the expression of UCHL1 and CALD1 enhances the tissue specificity of the cells, while the expression of TXNIP and DDIT3 decreases the tissue specificity of cells. Therefore, as shown in Supplementary Fig. S17d, cluster 0 can be defined as cells with ‘high tissue specificity’, and cluster 1 can be defined as cells with ‘low tissue specificity’. This observation suggests that scMCs can more comprehensively mine the single-cell multi-omics data by giving different clusterings

### 3.5 Ablation study and parameter sensitivity analysis

To study the contribution factors of scMCs, we introduce four variants: w/oAtt, w/oDiscriminator, w/oCL, and w/oZB, which separately disregard the attention layer, omics-label discriminator, contrastive learning, and ZINB loss and Bernoulli loss. Supplementary Fig. S18 reveals the average NMI and ARI values of scMCs and its variants. We observe that scMCs outperforms its variants by a clear margin, which confirms that attention layer, omics-label, contrastive learning mechanism, and generative decoder indeed contribute to the quality of cell clustering. More analyses are given in Supplementary Section S4. Taking CellMix as an example, we also conduct different experiments to evaluate the parameter sensitivity of scMCs. The details are reported in Supplementary Figs S19–S21 in Supplementary Section S5. In general, scMCs can show better clustering performance without much effort to adjust parameters.

## 4 Conclusion

In this article, we propose scMCs for single-cell multi-omics data fusion, cell clustering, and multiple clusterings. scMCs extracts the individual and shared features of multi-omics data and fuses them into an informative co-embedding representation for clustering and imputation. Moreover, scMCs can comprehensively mine multi-omics data by projecting the co-embedding representation into different salient subspaces to generate different and meaningful alternative clusterings. Experimental results show that scMCs can achieve superior and competitive performance in cell clustering and data imputation. More importantly, scMCs finds out multiple clustering structures with diversity and quality, which provide new insights of understanding the diverse roles of cells from different perspectives. How to couple data fusion and multiple clustering mining into a unified method and simplifying scMCs with fewer parameters (ideally parameter-free) are two future pursues for single-cell data multiple clusterings.

## Supplementary Material

btad133_Supplementary_Data

## Data Availability

The data underlying this article are available in Gene Expression Omnibus, at https://www.ncbi.nlm.nih.gov/geo/.
