# Supplementary material for: scMCs: a framework for single-cell multi-omics data integration and multiple clusterings
Source: Bioinformatics. 2023 Mar 17;39(4):btad133. doi: 10.1093/bioinformatics/btad133 (PMC10070040; doi:10.1093/bioinformatics/btad133)
Supplement: btad133_Supplementary_Data [file btad133_supplementary_data.pdf]

scMCs: a framework for single cell multi-omics data integration and multiple clusterings

Liangrui Ren, Jun Wang, Zhao Li, Qingzhong Li, Guoxian Yu

February 21, 2023

1 Performance of baselines on CellMix, PBMC_3K, Mouse_skin and AdBrain

We visualize the clustering results of baselines on CellMix, PBMC_3K, Mouse_skin and AdBrain in Fig. S1 ∼ Fig. S4. Fig. S5 ∼ Fig. S8 provide the visualization and clustering results of different methods on each raw and imputed scRNA-seq data, while Fig. S9 ∼ Fig. S12 show the visualization and clustering results of each method on raw and imputed scATAC data. According to these results and those in the main text, we can see that scMCs can obtain clearer visualization results than the baseline methods in most cases. In addition, the clustering results of scMCs and of other imputed methods against the ground truth of CellMix, PBMC_3K, Mouse_skin and AdBrain indicate the scMCs achieves the best imputation and clustering results.

2 Downstream analysis on imputed AdBrain

Fig. S13 reports the downstream analysis results on imputed Adbrain. We can find that the clusters obtained using the imputed data are more compact, and the boundaries between the clusters are clearer. Moreover, we create the gene activity matrix based on the imputed scATAC data, and accurately identify the differentially expressed genes of each cell cluster. In addition, we also find the differentially accessible peaks using the imputed scATAC data, and observe that the peaks are significantly different among clusters, which indicates the specific accessibility in heterogeneous cell types.

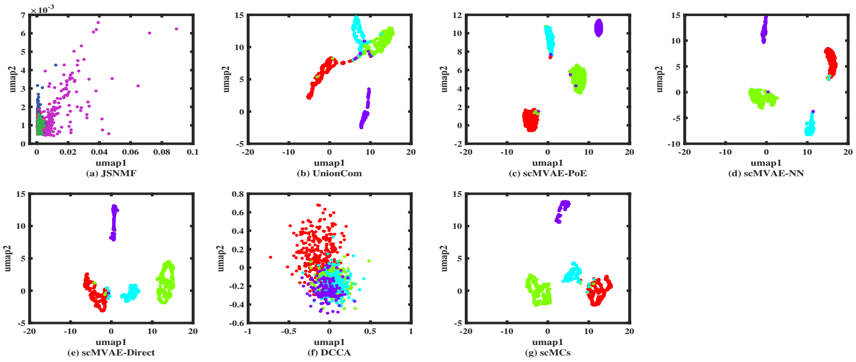

Figure S1: Cell clustering visualization of each method on CellMix. (a) JSNMF; (b) UnionCom; (c) scMVAE-PoE; (d) scMVAE-NN; (e) scMVAE-Direct; (f) DCCA; (g) scMCs.

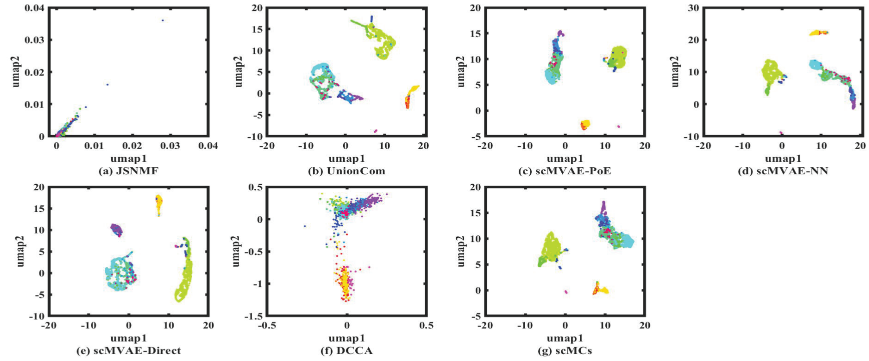

Figure S2: Cell clustering visualization of each method on PBMC_3K. (a) JSNMF; (b) UnionCom; (c) scMVAE-PoE; (d) scMVAE-NN; (e) scMVAE-Direct; (f) DCCA; (g) scMCs.

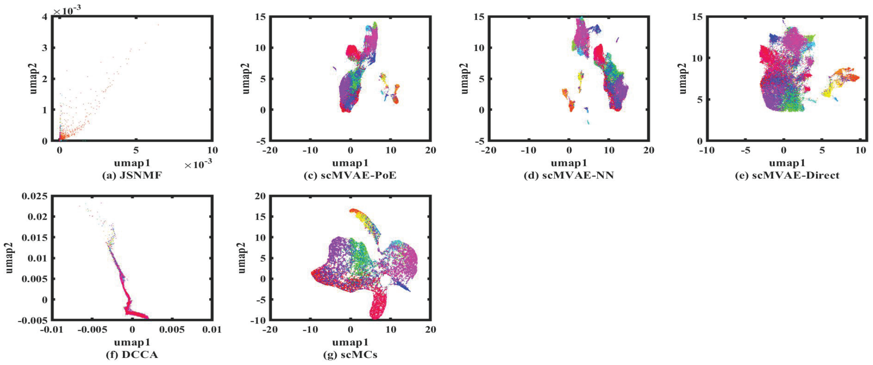

Figure S3: Cell clustering visualization of each method on Mouse_skin. (a) JSNMF; (b) UnionCom; (c) scMVAE-PoE; (d) scMVAE-NN; (e) scMVAE-Direct; (f) DCCA; (g) scMCs.

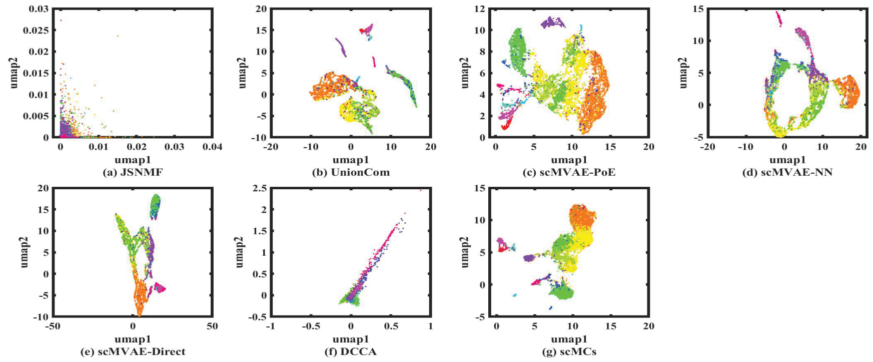

Figure S4: Cell clustering visualization of each method on AdBrain. (a) JSNMF; (b) UnionCom; (c) scMVAE-PoE; (d) scMVAE-NN; (e) scMVAE-Direct; (f) DCCA; (g) scMCs.

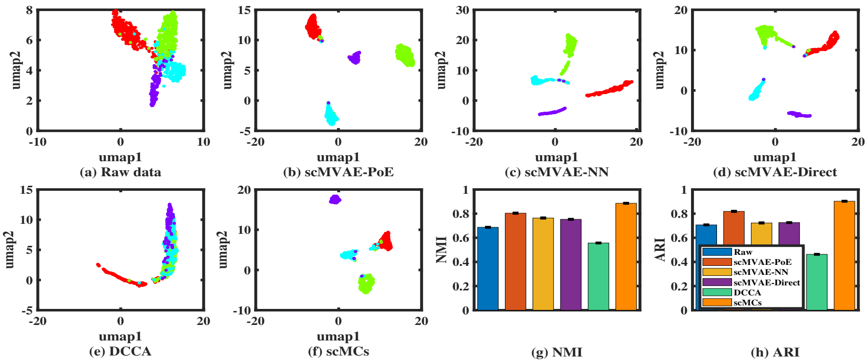

Figure S5: Cell clustering visualization of each method on raw and imputed CellMix scRNA-seq data. (a) Raw data; (b) scMVAE-PoE; (c) scMVAE-NN; (d) scMVAE-Direct; (e) DCCA; (f) scMCs; (g) NMI values; (h) ARI values.

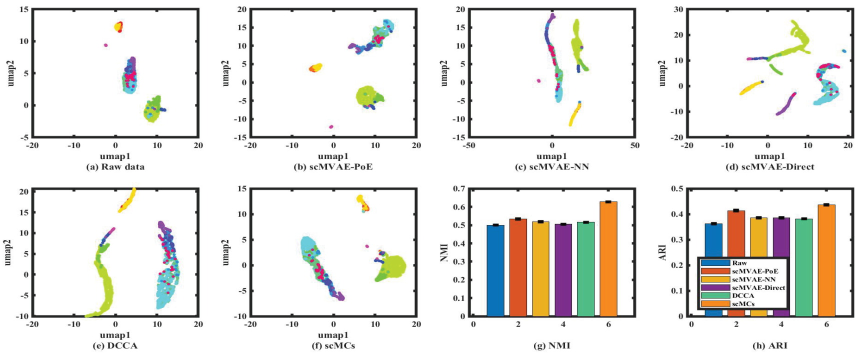

Figure S6: Visualization and clustering performance of imputed methods on raw and imputed PBMC_3K scRNA-seq data. (a) Raw data; (b) scMVAE-PoE; (c) scMVAE-NN; (d) scMVAE-Direct; (e) DCCA; (f) scMCs; (g) NMI; (h) ARI.

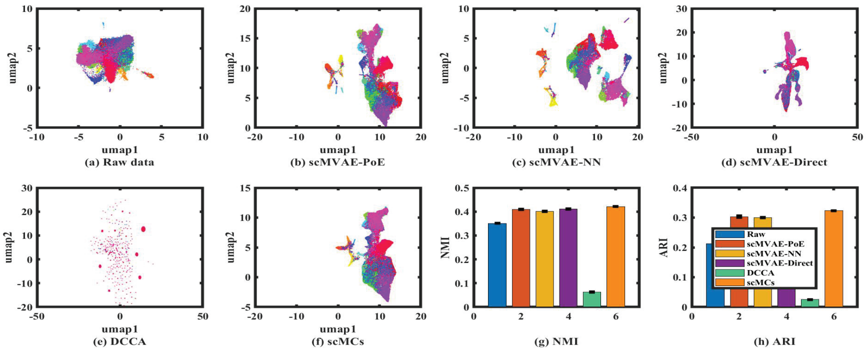

Figure S7: Visualization and clustering performance of imputed methods on raw and imputed Mouse_skin scRNA-seq data. (a) Raw data; (b) scMVAE-PoE; (c) scMVAE-NN; (d) scMVAE-Direct; (e) DCCA; (f) scMCs; (g) NMI; (h) ARI.

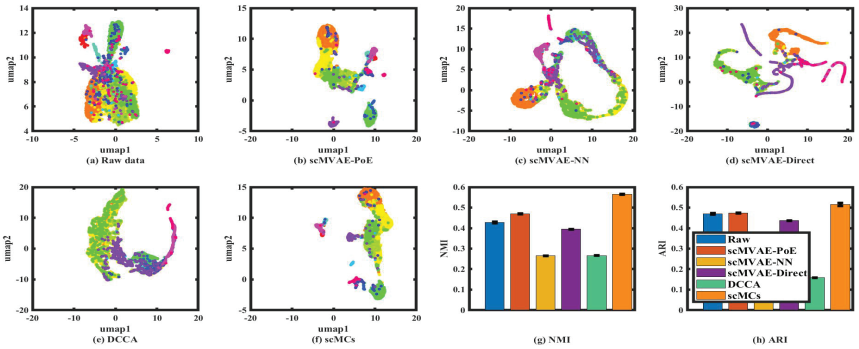

Figure S8: Visualization and clustering performance of imputed methods on raw and imputed AdBrain scRNA-seq data. (a) Raw data; (b) scMVAE-PoE; (c) scMVAE-NN; (d) scMVAE-Direct; (e) DCCA; (f) scMCs; (g) NMI; (h) ARI.

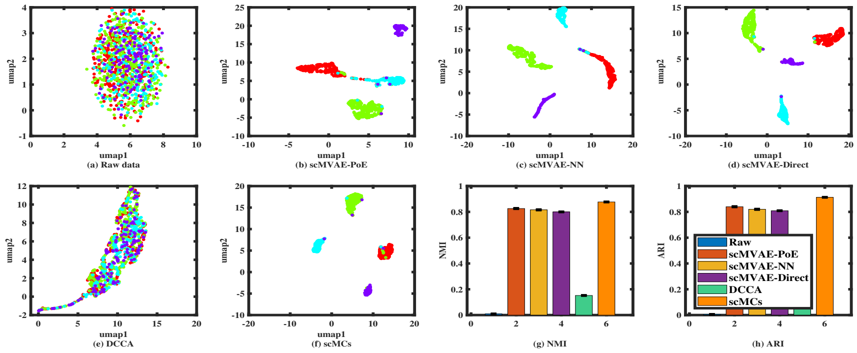

Figure S9: Cell clustering visualization of each method on raw and imputed CellMix scATAC data. (a) Raw data; (b) scMVAE-PoE; (c) scMVAE-NN; (d) scMVAE-Direct; (e) DCCA; (f) scMCs; (g) NMI values; (h) ARI values.

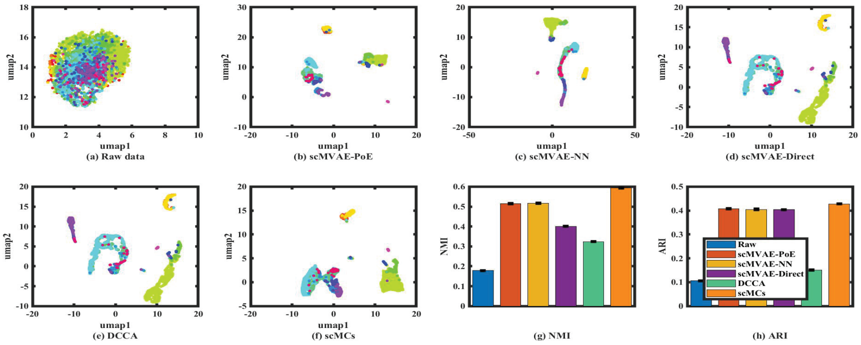

Figure S10: Visualization and clustering performance of imputed methods on raw and imputed PBMC_3K scATAC data. (a) Raw data; (b) scMVAE-PoE; (c) scMVAE-NN; (d) scMVAE-Direct; (e) DCCA; (f) scMCs; (g) NMI; (h) ARI.

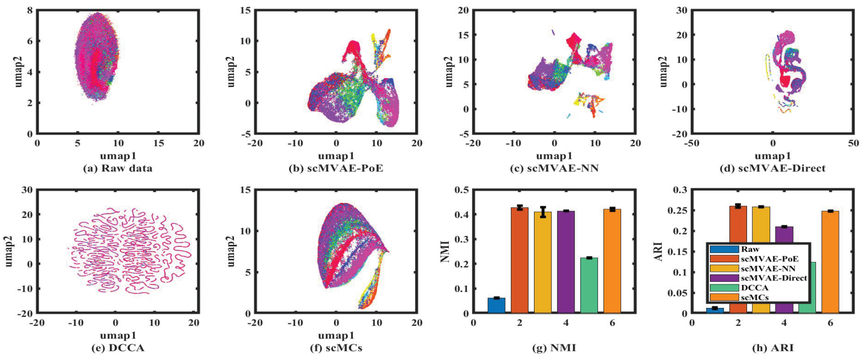

Figure S11: Visualization and clustering performance of imputed methods on raw and imputed Mouse_skin scATAC data. (a) Raw data; (b) scMVAE-PoE; (c) scMVAE-NN; (d) scMVAE-Direct; (e) DCCA; (f) scMCs; (g) NMI; (h) ARI.

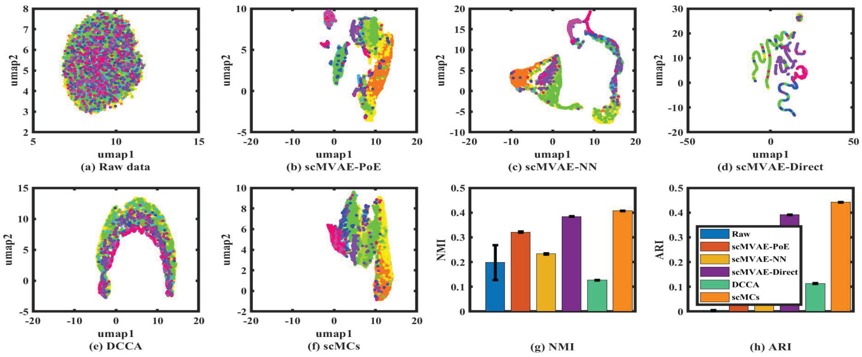

Figure S12: Visualization and clustering performance of imputed methods on raw and imputed AdBrain scATAC data. (a) Raw data; (b) scMVAE-PoE; (c) scMVAE-NN; (d) scMVAE-Direct; (e) DCCA; (f) scMCs; (g) NMI; (h) ARI.

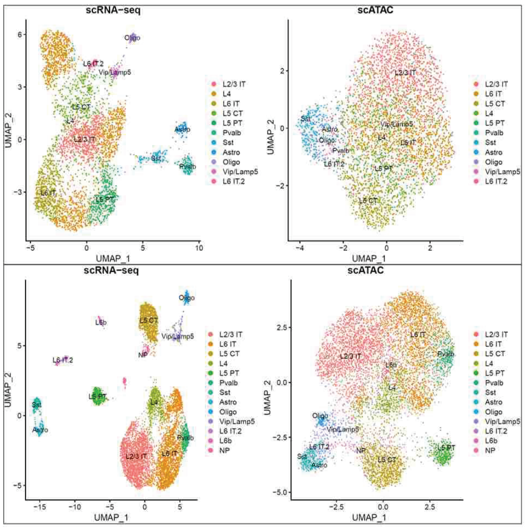

(a)

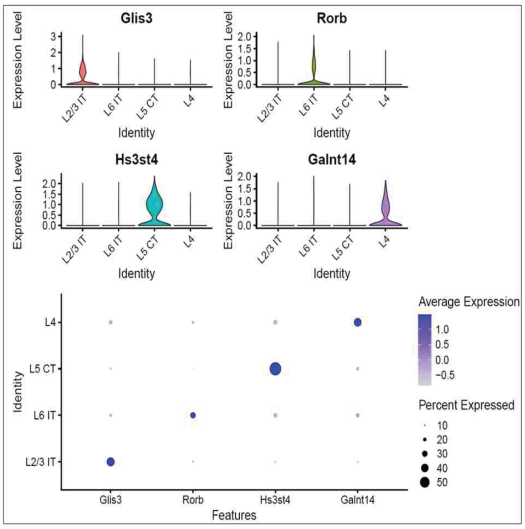

(b)

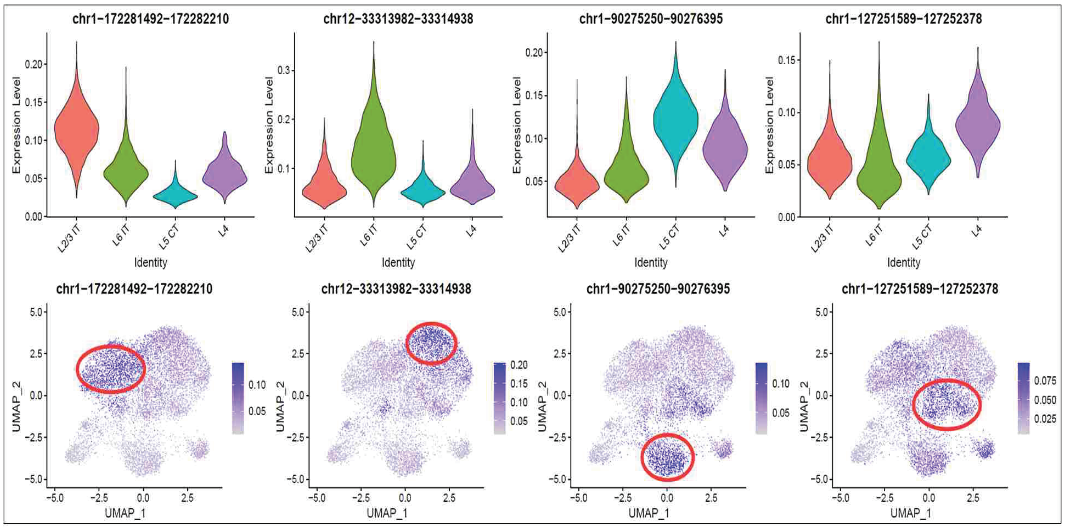

(c)

Figure S13: Downstream analysis on the imputed AdBrain. (a) Cell type annotation and clustering on raw and imputed AdBrain; (b) Selected differentially expressed genes on gene activity matrix; (c) Identified differentially accessible peaks based on peak counts of imputed AdBrain.

3 Downstream analysis of multiple clusterings generated from CellMix

Fig. S14 reports the diversity (1-NMI, 1-JI) of scMCs on CellMix, PBMC_3K and AdBrain. Fig. S15 $\sim$ Fig. S17 report the downstream analysis results of multiple clusterings generated from CellMix, where Fig. S15 reveals the results based on the ground truth $\mathcal{C}_t$, Fig. S16 reveals the results of $\mathbf{O}_1$ based on $\mathcal{C}_1$, and Fig. S17 shows the results of $\mathbf{O}_2$ based on $\mathcal{C}_2$. Based on these results, we can find that scMCs discovers two clusterings with diversity and quality, where $\mathcal{C}_1$ is a clustering about cell types, while $\mathcal{C}_2$ divides cells into two clusters based on whether they are tissue-specific.

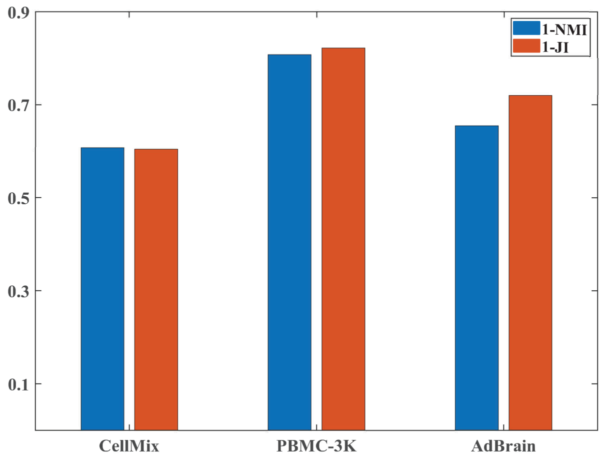

Figure S14: Diversity(↑) between $\mathcal{C}_1$ and $\mathcal{C}_2$ generated by scMCs.

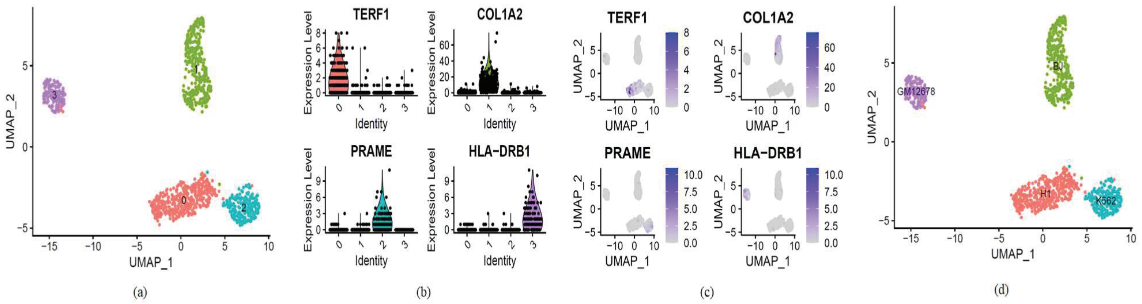

Figure S15: Downstream analysis of CellMix and $\mathcal{C}_t$. (a) Cell clustering visualization based on $\mathcal{C}_t$; (b) and (c) Identified cell marker genes; (d) Cell type annotation based on the marker genes.

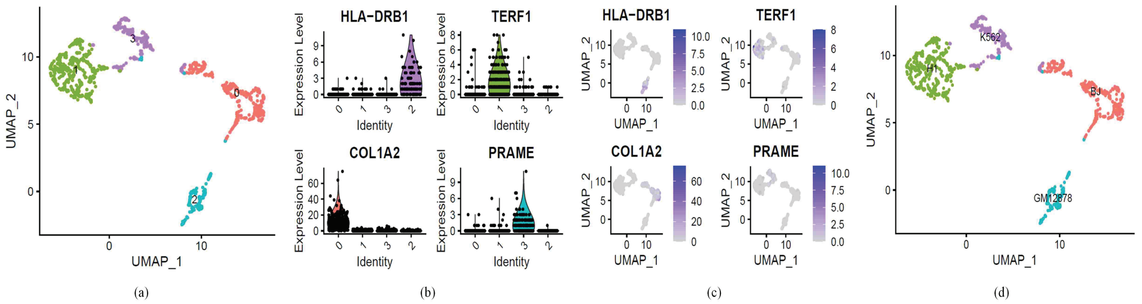

Figure S16: Downstream analysis of $\mathbf{O}_1$ and $\mathcal{C}_1$. (a) Cell clustering visualization based on $\mathcal{C}_1$; (b) and (c) Identified cell marker genes; (d) Cell type annotation based on the marker genes.

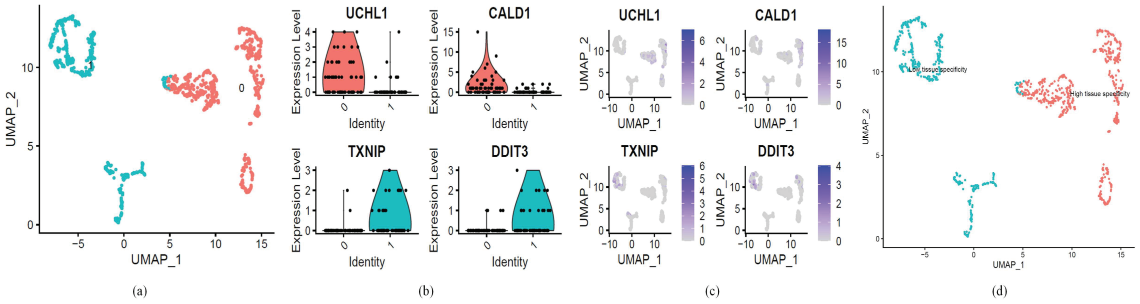

Figure S17: Downstream analysis of $\mathbf{O}_2$ and $\mathcal{C}_2$. (a) Cell clustering visualization based on $\mathcal{C}_2$; (b) and (c) Identified cell marker genes; (d) Cell type annotation based on the marker genes.

4 Ablation study

To study the contribution factors of scMCs, we introduce four variants: w/oAtt, w/oDiscriminator, w/oCL and w/oZB, which separately disregard the attention layer, omics-label discriminator, contrastive learning, and ZINB loss and Bernoulli loss. Fig. S18 reveals the average NMI and ARI values of scMCs and its variants.

From Fig. S18, we observe that scMCs outperforms its variants by a clear margin, which confirms that attention layer, omics-label, contrastive learning mechanism, and generative decoder indeed contribute to the quality of cell clustering. Among them, w/oZB usually has the lowest results, which suggests the importance of ZINB loss and Bernoulli loss on guiding cell clustering and data imputation. The contribution of contrastive learning is often larger than the attention layer and omics-label discriminator. This is because the cross-omics shared features learned via contrastive learning are important for the consistent clustering with respect to cell types. The individual features of different omics are also important for a high quality clustering, but only the individual features may lead to a meaningless clustering. In fact, we find that the individual features are more important for generating alternative clusterings with diversity.

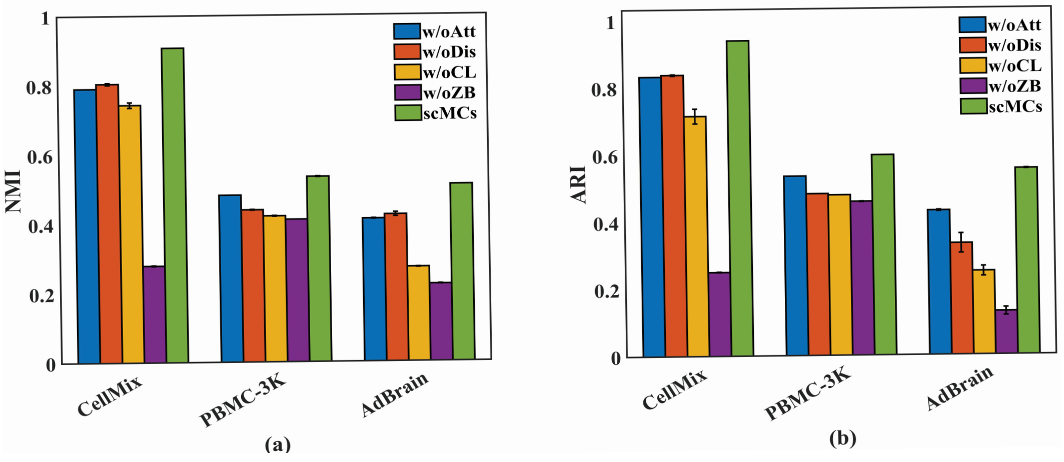

Figure S18: The performance of scMCs and its variants.

5 Parameter sensitivity analysis

Taking CellMix as an example, we conduct different experiments to evaluate the sensitivity of scMCs to different parameters combinations. The details are as follows:

5.1 Sensitivity analysis of $\{\alpha_1, \alpha_2, \alpha_3\}$

We first measure the impact of three parameters, $\{\alpha_1, \alpha_2, \alpha_3\}$ in Eq. (17) on the clustering performance of scMCs, which balance the $\mathcal{L}_{Ber}$, $\mathcal{L}_{dis}$ and $\mathcal{L}_{cl}$, respectively. As shown in Fig. S19, the performance of scMCs is relatively stable when $\alpha_1 = 0.0001$. With the increase of α_1, the clustering performance of scMCs fluctuated significantly. This is because scATAC data has high dimensionality and sparsity, and a larger $\mathcal{L}_{Ber}$ loss will affect the optimization of other modules of scMCs, resulting in suboptimal clustering performance. In addition, we also find that scMCs is insensitive to different combinations of α_2 and α_3, especially when $\alpha_1 = 0.0001$. It can be found that the clustering performance of scMCs is poor when $\alpha_2 < 0.01$ and $\alpha_3 < 0.1$. This is because a smaller α_2 and α_3 weaken the constraint of label prediction loss and cross-omics contrastive learning loss, resulting in insufficient learning of omics-specific and consistent features.

5.2 Sensitivity analysis of $\{\lambda_x, \lambda_y\}$

Then, we further evaluate the impact of $\{\lambda_x, \lambda_y\}$ in Eq. (8), where $\{\lambda_x, \lambda_y\}$ are the weight parameters of omics specific features learned from scRNA-seq and scATAC data, respectively. From Fig. S20, it can be found that the clustering performance of scMCs is better when $\lambda_x \in [0.1, 1]$ and $\lambda_y \in [0.001, 0.01]$. This is because the dimensionality and sparsity of scATAC data are much higher than those of scRNA-seq data, resulting in lower quality of $\mathbf{Z}_{gY}$ than $\mathbf{Z}_{gX}$, even though we have utilized the Bernoulli distribution to model the dropout events in scATAC data. Therefore, to ensure learning a more discriminative and accurate co-embedding matrix $\mathbf{Z}_I$, we set $\lambda_x = 1$, and $\lambda_y = 0.01$.

5.3 Sensitivity analysis of $\{\beta_1, \beta_2\}$

We also conduct experiments on CellMix to analyze the diversity (1-NMI) and quality (SI) of scMCs with different combinations of β_1 and β_2, and report the results in Fig. S21. We can see that the diversity and quality of scMCs are poor when $\beta_1 \leq 0.1$ and $\beta_2 \leq 0.1$. This is because the smaller β_1 and β_2 weaken the constraint of HSIC and KL divergence on the diversity and quality, causing scMCs to focus on reconstruction loss but fail to produce significant different clusterings.

To ensure the fairness of the experiment, we set the parameters of each baseline as follows:

Table S1: Parameter settings of each baseline on benchmark datasets.

	CellMix	PBMC_3K	Mouse_skin	AdBrain
JSNMF	$\eta = 0.5$<br>$\alpha = 10$<br>$\gamma = 10$	$\eta = 0.5$<br>$\alpha = 1$<br>$\gamma = 10$	$\eta = 0.5$<br>$\alpha = 10$<br>$\gamma = 10$	$\eta = 0.5$<br>$\alpha = 1$<br>$\gamma = 10$
UnionCom	$\alpha = 0.1$	$\alpha = 0.1$	$\alpha = 0.1$	$\alpha = 0.1$
scMVAE	$\lambda_1 = 0.1$<br>$\lambda_2 = 1$<br>$\beta = 0.5$	$\lambda_1 = 0.1$<br>$\lambda_2 = 0.1$<br>$\beta = 0.5$	$\lambda_1 = 0.1$<br>$\lambda_2 = 0.01$<br>$\beta = 0.5$	$\lambda_1 = 0.0001$<br>$\lambda_2 = 0.1$<br>$\beta = 0.5$
DCCA	$\lambda = 0.5$<br>$\beta = 0.1$	$\lambda = 0.5$<br>$\beta = 0.01$	$\lambda = 0.5$<br>$\beta = 0.01$	$\lambda = 0.1$<br>$\beta = 0.1$
scMCs	$\lambda_x = 1$<br>$\lambda_y = 0.01$<br>$\alpha_1 = 0.0001$<br>$\alpha_2 = 0.001$<br>$\alpha_3 = 0.01$<br>$\beta_1 = 1$<br>$\beta_2 = 1$	$\lambda_x = 1$<br>$\lambda_y = 0.01$<br>$\alpha_1 = 0.01$<br>$\alpha_2 = 0.001$<br>$\alpha_3 = 0.001$<br>$\beta_1 = 1$<br>$\beta_2 = 1$	$\lambda_x = 0.01$<br>$\lambda_y = 0.001$<br>$\alpha_1 = 0.01$<br>$\alpha_2 = 0.0001$<br>$\alpha_3 = 0.001$<br>−<br>−	$\lambda_x = 0.1$<br>$\lambda_y = 0.001$<br>$\alpha_1 = 0.01$<br>$\alpha_2 = 0.001$<br>$\alpha_3 = 0.001$<br>$\beta_1 = 0.1$<br>$\beta_2 = 1$

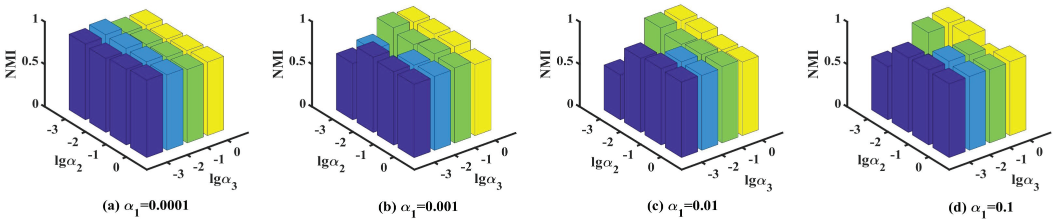

Figure S19: The clustering performance of scMCs under different combinations of α_1, α_2 and α_3.

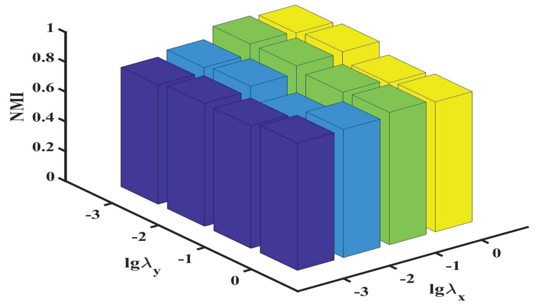

Figure S20: The clustering performance of scMCs under different combinations of $\{\lambda_x, \lambda_y\}$.

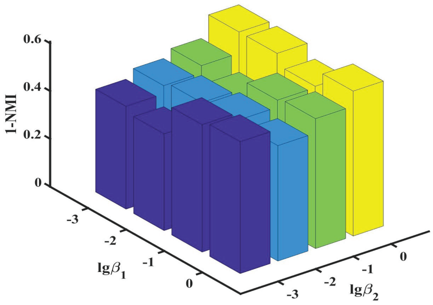

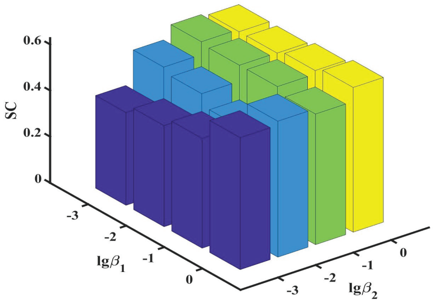

Figure S21: Diversity (↑) and quality (↓) of scMCs under different combinations of $\{\lambda_x, \lambda_y\}$.
